# Oncological Outcomes Following Computer-Aided Reconstructive Jaw Surgery

**DOI:** 10.3390/cmtr18010008

**Published:** 2025-01-05

**Authors:** John M. Le, John Hofheins, Myra Rana, Jay Ponto, Anthony B. Morlandt, Yedeh P. Ying

**Affiliations:** 1 Department of Oral and Maxillofacial Surgery, University of Alabama at Birmingham, Birmingham, AL 35233, USA; jhofheins@uabmc.edu (J.H.); jponto@uabmc.edu (J.P.); morlandt@uab.edu (A.B.M.); yying@uab.edu (Y.P.Y.); 2 School of Dentistry, University of Alabama at Birmingham, Birmingham, AL 35233, USA; ranamyra19@gmail.com

**Keywords:** oral oncology, virtual surgery, reconstructive surgery, oral cancer, local recurrence

## Abstract

The purpose of this study was to analyze computer-aided surgical planning (CAS) and margin status following oncological reconstructive surgery of the jaws. A retrospective study was conducted on patients who underwent microvascular reconstructive surgery from 2014 to 2021. The predictor variable was the use of CAS. The primary and secondary outcomes were histopathological bone margin status, local recurrence, and disease-free survival (DFS). Covariates included demographic, operative, pathological, and clinical outcomes. Thirty-five CAS and fifty-two non-CAS subjects were included for analysis. Demographic characteristics such as age, sex, and comorbidities were comparable between the study groups, with all *p*-values > 0.05. For operative variables, the osteocutaneous radial forearm flap was more commonly used in the non-CAS group (34.6%) compared to the CAS group (2.9%) (*p* < 0.01). The mean follow-up period was shorter in the CAS group (31.9 months) than in the non-CAS group (42.6 months) (*p* < 0.01). CAS was not associated with margin status (*p* = 0.65) or local recurrence (*p* = 0.08). DFS was comparable between the study groups (*p* = 0.74). Bone margin involvement was not associated with any covariates. The use of CAS in oncological reconstructive jaw surgery was not associated with increased bone margin involvement.

## 1. Introduction

Surgical resection margins in oncological surgery and microvascular reconstruction of the jaws are major prognostic indicators for recurrence and survival in patients with oral cavity cancer. An involved margin is an independent predictor for local recurrence and overall survival [1,2,3,4,5]. Furthermore, a positive margin is considered to be an adverse factor that would necessitate re-excision and/or adjuvant radiotherapy according to the National Comprehensive Cancer Network (NCCN) guidelines and the literature [6,7].

Since its inception and application in orthognathic surgery, computer-aided surgical planning is now used across multiple maxillofacial procedures, including trauma, temporomandibular joint replacement, and oncological and microvascular reconstructive surgery [8,9,10,11,12,13]. Since it was first described by Hirsch et al. for head and neck reconstruction, CAS is now incorporated into most microvascular reconstructive jaw surgeries [14,15,16]. Because the application of CAS for surgical planning and for model and cutting guide fabrication requires additional time from diagnosis to treatment, there is a valid concern for tumor growth and risk for a close or involved margin at the time of surgery. While this concern exists, there have been studies that show that R0 resection is achievable using CAS for benign and malignant diseases [17,18,19,20].

The purpose of this study was to determine whether the use of computer-aided surgery (CAS) in oncological microvascular reconstructive surgery of the jaws is associated with surgical margin integrity, local recurrence, and survival outcomes in patients with oral cavity squamous-cell carcinoma. The authors believe that using CAS does not compromise the margin integrity in oncological jaw resection or affect survival outcomes.

## 2. Materials and Methods

### 2.1. Study Design and Sample

**Figure 1:**
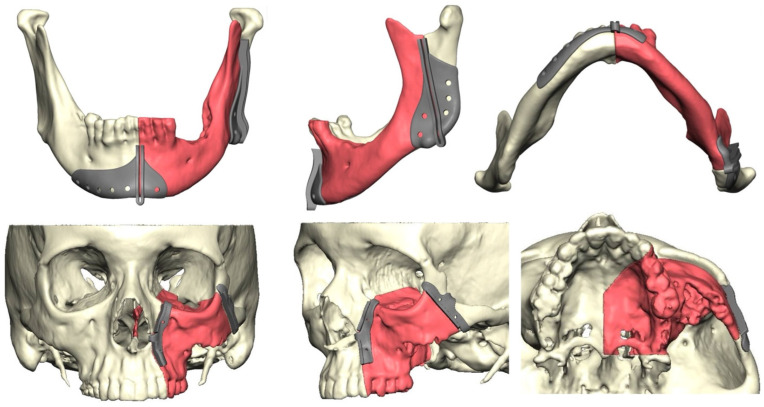
This figure shows the design and location of the predetermined resection margins using patient-specific surgical cutting guides for cases involving the mandible (**top row**) and maxilla (**bottom row**).

Following approval by the Institutional Review Board (IRB), a retrospective chart review of patients who underwent microvascular reconstructive surgery of the oral cavity from January 2014 to July 2021 was performed. Subjects with squamous-cell carcinoma associated with the maxilla or mandible, and who required a vascularized bone flap for reconstruction with at least 12 months of follow-up, were included in the study. Benign pathology, non-bone-containing vascularized flaps, and non-vascularized bone grafts were excluded from the study. The predictor variable was the use of CAS, which included virtual surgical planning using a third-party provider, KLS Martin North America (Jacksonville, FL, USA). Surgical resection in the CAS group was predetermined and included custom patient-specific cutting guides designed and manufactured by KLS Martin North America (Jacksonville, FL, USA) (Figure 1). In the non-CAS group, resection margins were determined intraoperatively by the ablative surgeon.

### 2.2. Covariates

Covariates included demographic variables such as age, sex, American Society of Anesthesiologists (ASA) score, body mass index (BMI), tobacco history (never or ever), and alcohol history (never or ever). Operative variables included the time from the initial clinic visit to treatment (days), jaw (maxilla or mandible), flap type used (osteocutaneous radial forearm flap (OC-RFFF) or fibular free flap (FFF)), procedure time (minutes), estimated blood loss (EBL), and flap bone segments used. Pathological variables included the tumor stage, nodal stage, cancer stage, presence of perineural invasion (PNI), presence of lymphovascular invasion (LVI), and presence of extranodal extension (ENE). Finally, outcome variables were measured for the most recent clinic visit (months), type of adjuvant therapy received, and clinical outcome. Clinical outcomes were categorized as no evidence of disease (NED), alive with disease (AWD), died of disease (DOD), or died of other causes (DOO). Disease-free survival (DFS) was measured as NED, alive without recurrence, or DOO at the most recent follow-up appointment.

### 2.3. Study Outcomes

The primary outcome was histologically positive bone margin status (yes or no) on the final pathology report. Positive soft tissue margins in the absence of a positive bone margin were not analyzed. Close margins were grouped into the negative margin status. A secondary outcome analyzed was local recurrence (yes or no) within five years following the primary treatment.

### 2.4. Statistical Analysis

Continuous data were presented as the mean with standard deviation (SD), and categorical data were presented as the number (percentage). Bivariate analyses were conducted through Student’s t-test and chi-squared tests. For unequal distribution or small sample sizes, a nonparametric Mann–Whitney U test or Fisher’s exact test was performed. Kaplan–Meier survival analysis was performed for DFS. All statistical tests were conducted using SPSS software version 28 (SPSS Inc., Chicago, IL, USA). For all tests, *p*-values of <0.05 were regarded as statistically significant.

## 3. Results

A total of 87 subjects met the criteria for analysis (35 CAS and 52 non-CAS). Associations between the CAS and non-CAS groups are shown in Table 1. Demographic variables were comparable between the study groups (*p* > 0.05). For operative variables, a significantly larger amount of OC-RFFF was used to reconstruct the jaw in the non-CAS group (34.6%) compared to the CAS group (2.9%) (*p* < 0.01). The time-lapse from diagnosis to treatment was comparable between the groups (CAS, 31.8 days vs. non-CAS, 27.8 days; *p* = 0.24). For clinical outcomes, the mean follow-up period was shorter in the CAS group (31.9 months) compared to the non-CAS group (42.6 months) (*p* < 0.01). A greater number of subjects had NED at the most recent clinic visit (88.6%) in the CAS group compared to the non-CAS group (59.6%). Additionally, 20% of subjects in the non-CAS group were AWD, compared to 12.2% in the CAS group (Table 1).

Regarding the primary outcome, one subject (1.1%) had a positive bone margin and was in the non-CAS group (Table 2 and Table 3). Positive soft tissue margins were recorded in three subjects (3.4%) and were not associated with a positive bone margin (Table 4). A total of 12 subjects (13.8%) had close soft tissue margins (<5 mm) without positive bone margins. A subgroup analysis showed that close margins had no association with pathological stage or tumor location.

All four subjects with positive margins had mandibular involvement and did not undergo re-excision. Re-excision was not performed due to reasons including subjects being medically unfit or the need for removal of the bone flap. Three subjects elected for adjuvant radiotherapy with or without concurrent chemotherapy. One subject declined adjuvant treatment and elected for observation. All four subjects had locoregional or distant metastasis within the study period. These findings are shown in Table 5.

Twenty-one subjects experienced local recurrence within 5 years of primary treatment. No demographic, operative, or pathological variables were found to be associated with local recurrence. Similar to margin status, the clinical outcome of NED was more prevalent in the group without local recurrence, while AWD was more common in the group with local recurrence. Associations between subjects with and without local recurrence are outlined in Table 6. CAS was not associated with local recurrence, with a *p*-value of 0.08 (Table 7).

**Figure 2:**
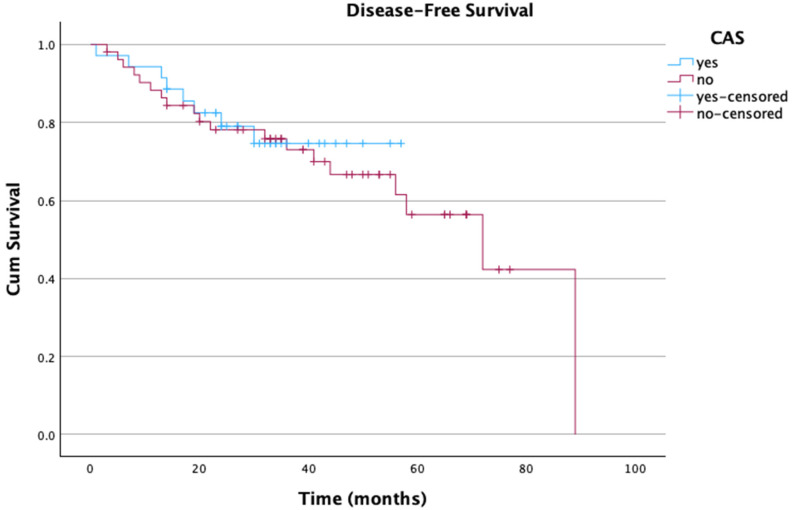
Kaplan–Meier survival curve comparing disease-free survival between study groups.

Finally, DFS was comparable between groups, with the CAS group having a mean of 46.8 months (standard error (SE) = 3.18, 95% CI [40.54 to 53.03]) and the non-CAS group having a mean of 60.7 months (SE = 5.24, 95% CI = [50.43 to 70.99]) (*p* = 0.74), as shown in Figure 2. No demographic, operative, or pathological variables were found to be associated with DFS in this study population.

## 4. Discussion

To the best of our knowledge, this is the largest retrospective analysis comparing resection margin status, local recurrence patterns, and disease-related outcomes in subjects undergoing microvascular reconstruction of the maxilla and mandible for oral squamous-cell carcinoma. Our findings indicate that CAS provided comparable oncological margins to non-CAS cases and yielded similar clinical outcomes. Furthermore, the time to treatment was also comparable between study groups and occurred within one month.

Pu et al. [17] conducted a retrospective study comparing CAS and non-CAS groups in terms of resection margins, recurrence patterns, and survival outcomes. In their study of 66 subjects, 37 were in the CAS group and 29 in the non-CAS group, with all CAS planned in-house by the surgical team. The resection margins were comparable between study groups, with positive margins noted in 8.1% of the CAS group and 17.1% of the non-CAS group (*p* = 0.39). Notably, all bone margins were negative, and no changes in segmental mandibulectomy margins or fibula planning were reported [17]. Similarly, Knitschke et al. [19] compared virtual surgical planning (VSP) and patient-specific implants (PSIs) versus non-VSP and hand-bent stock plates in 104 subjects undergoing fibular free flap reconstruction of the jaws. They found no statistically significant differences in bone and soft tissue resection margins between the study groups, with positive bone margins in 4.9% of VSP cases and 3.2% of non-VSP cases (*p* = 0.52), and with positive soft tissue margins in 12.2% of VSP cases and 6.3% of non-VSP cases (*p* = 0.47). Barry et al. [20] also reported that VSP cases did not increase the risk of involved or close bone margins (*p* = 0.49) or soft tissue margins (*p* = 0.22). In our study, bone margins were positive in zero CAS cases and 1.1% of non-CAS cases (*p* = 1.00), while soft tissue margins were positive in 2.9% of CAS cases and 3.8% of non-CAS cases (*p* = 1.00). Among the four subjects with positive margins, one declined adjuvant therapy, and the remainder received adjuvant radiotherapy with or without concurrent chemoradiotherapy.

In this study, CAS was not found to be associated with local recurrence (*p* = 0.08). Margin status was not found to be associated with local recurrence in this study cohort. Of the four subjects with a positive margin, two (50%) experienced local recurrence. These findings align with the existing literature, where margin status consistently emerges as an independent predictor of local recurrence [3,4,5]. CAS was also found to be unrelated to disease-free survival, consistent with previous studies [17,21].

When employing third-party sources for CAS, Succo et al. [22] reported an average time of 15 ± 3 days for completing cutting guides and customized plates for mandibular reconstruction with a fibular free flap. Similar, Kirke et al. [23] noted comparable timelines for virtual surgical planning sessions and the fabrication of guides and models for complex mandibular defect reconstructions. Notably, VSP and PSI cases necessitate involvement from third parties, thereby affecting the time from diagnosis to surgery compared to non-VSP cases [19]. The time interval from the initial clinic visit to surgery was shown to be significantly longer in the VSP group (47.2 ± 24.5 days) compared to the non-VSP group (35.7 ± 18.6 days) (*p* = 0.008). In our experience, it takes approximately 14 days for an approved surgical plan to complete the cutting guides and surgical model. Our team primarily utilizes stock titanium plates bent to the surgical models for the majority of oncology cases, reserving custom, patient-specific plates for benign cases and trauma. While Pu et al. [17] noted that CAS cases planned at their institution generally took less than 2 weeks, they did not compare the time from the initial clinic visit to surgery between their study groups. In our study, the time interval was comparable between the CAS group (31.8 ± 17.9 days) and the non-CAS group (27.8 ± 13.7 days) (*p* = 0.24). This period is notably shorter than that reported by Knitschke et al. [19]. However, both ranges are within the critical threshold of 60 days, which has been statistically associated with increased mortality risk based on findings from Murphy et al.’s [24] analysis of 51,655 patients using the National Cancer Data Base. Notably, there is no unified national target for treatment times for head and neck cancers across countries. For instance, Barry et al. [20] reported similar treatment times of 65 ± 30 days in the non-VSP group and 59 ± 16 days in the VSP group (*p* = 0.37), although these ranges differ significantly from those reported in the United States of America.

Achieving negative bone margins using computer-aided surgical planning is important, but it is not as challenging as achieving negative soft tissue margins. Generally, resection guides are planned at least 10 mm from the tumor identified on radiographic imaging, although margins greater than 10 mm have also been reported to account for potential tumor growth from the initial clinic visit to the day of surgery [21,25,26,27]. However, thin-section CT, used to determine surgical resection margins, has imperfect sensitivity and specificity in identifying bone invasion. For instance, Struckmeier et al. [28] found that thin-section (1 mm slices) pre-operative CT exhibited 77% sensitivity, 82% specificity, 47% positive predictive value (PPV), and 90% negative predictive value (NPV) for identifying bone invasion in oral squamous-cell carcinoma (OSCC). Combining a thorough clinical examination with multiple diagnostic imaging methods may enhance diagnostic accuracy. In our practice, surgical resection margins are planned at least 1 cm from the tumor front as identified in imaging studies (CT and/or MRI using 1 mm slices). These margins are adjusted to accommodate potential tumor growth by the time of surgery, or for reconstructive purposes to avoid transferring vascularized bone segments of less than 2 cm.

Limitations to this study include its sample size and retrospective design. The low number of cases with positive margins and local recurrence may have reduced this study’s statistical power. A larger sample size could identify clinical characteristics such as tumor stage or histopathological traits that are adverse prognostic factors for positive margins, local recurrence, and disease-free survival. In our study, margin status was a treated as a binary variable (yes or no), and we did not differentiate close margins (<5 mm) due to the small sample size, which would have further reduced the statistical power. While demographic and clinicopathological variables were balanced between the comparison groups, future prospective randomized controlled trials are needed to validate our findings. Nevertheless, our study sample is larger compared to the existing retrospective studies and further supports the idea that CAS for predetermined resection margins is oncologically sound, without compromising intraoperative time, margin status, or local recurrence.

## 5. Conclusions

The use of computer-aided surgical planning for oncological surgery and microvascular reconstruction of the jaw remains a comparable treatment option to conventional non-CAS techniques without compromising margin status, local recurrence, and disease-free survival.

## Figures and Tables

**Table 1:** Associations between study variables and CAS.

Study Variable	CAS n = 35	Non-CAS n = 52	*p*-Value
Age (years), SD	64.5 ± 9.4	63.2 ± 11.7	0.57
Sex, (%)			0.68
Female	15 (42.9)	20 (38.5)	
Male	20 (57.1)	32 (61.5)	
ASA class, (%)			0.34
2	0 (0)	2 (3.8)	
3	32 (91.4)	48 (92.3)	
4	3 (8.6)	2 (3.8)	
BMI, SD	26.5 ± 5.1	25.9 ± 5.4	0.62
Tobacco status, (%)			0.26
Current/former	18 (51.4)	33 (63.5)	
Never	17 (48.6)	19 (36.5)	
Alcohol status, (%)			0.62
Current/former	19 (54.3)	31 (59.6)	
Never	16 (45.7)	21 (40.4)	
Time to treatment (days), SD	31.8 ± 17.9	27.8 ± 13.7	0.24
Jaw, (%)			0.35
Maxilla	3 (8.6)	9 (17.3)	
Mandible	32 (91.4)	43 (82.7)	
Flap, (%)			<0.01 *
OC-RFFF	1 (2.9)	18 (34.6)	
FFF	34 (97.1)	34 (65.4)	
Procedure time (minutes), SD	598.8 ± 73.8	573.2 ± 92.8	0.87
EBL, SD	428.6 ± 226.6	489.6 ± 339.4	0.36
Bone segments, (%)			0.31
1	12 (34.3)	19 (36.5)	
2	18 (51.4)	21 (40.4)	
3	4 (11.4)	12 (23.1)	
4	1 (2.9)	0 (0)	
Tumor stage, (%)			0.41
pT_1_	1 (2.9)	7 (13.5)	
pT_2_	5 (14.3)	6 (11.5)	
pT_3_	2 (5.7)	3 (5.8)	
pT_4_	27 (77.1)	36 (69.2)	
Nodal stage, (%)			0.84
pN_0_	24 (68.6)	34 (65.4)	
pN_1_	4 (11.4)	8 (15.4)	
pN_2_	6 (17.1)	7 (13.5)	
pN_3_	1 (2.9)	3 (5.8)	
Stage			0.25
I	1 (2.9)	7 (13.5)	
II	4 (11.4)	2 (3.8)	
III	2 (5.7)	6 (11.5)	
IV	27 (77.1)	35 (67.3)	
IVb	1 (2.9)	2 (3.8)	
PNI present	7 (20)	17 (32.7)	0.19
LVI present	7 (20)	10 (19.2)	0.93
ENE present	3 (8.6)	4 (7.8)	1.00
Adjuvant therapy			0.93
None or declined	9 (25.7)	13 (25)	
Radiotherapy	21 (60)	30 (57.7)	
Chemoradiotherapy	5 (14.3)	9 (17.3)	
Follow-up (months)	31.9 ± 12	42.6 ± 21.2	<0.01 *
Clinical outcome			0.02 *
NED	31 (88.6)	31 (59.6)	
AWD	4 (11.4)	11 (21.2)	
DOD	0 (0)	5 (9.6)	
DOO	0 (0)	5 (9.6)	

* Statistically significant (*p* < 0.05).

**Table 2:** Associations between study variables and bone margin status.

Study Variable	Positive Margin n = 1	Negative Margin n = 86	*p*-Value
Age (years), SD	63	63.7 ± 10.8	0.95
Sex, (%)			1.00
Female	0 (0)	35 (40.7)	
Male	1 (100)	51 (59.3)	
ASA class, (%)			0.96
2	0 (0)	2 (2.3)	
3	1 (100)	79 (91.9)	
4	0 (0)	5 (5.8)	
BMI, SD	20.5	26.2 ± 5.2	0.28
Tobacco status, (%)			1.00
Current/former	1 (100)	50 (58.1)	
Never	0 (0)	36 (41.9)	
Alcohol status, (%)			1.00
Current/former	1 (100)	49 (57)	
Never	0 (0)	37 (43)	
Time to treatment (days), SD	8	29.6 ± 15.4	0.17
Jaw, (%)			1.00
Maxilla	0 (0)	12 (14)	
Mandible	1 (100)	74 (86)	
Flap, (%)			1.00
OC-RFFF	0 (0)	19 (22.1)	
FFF	1 (100)	67 (77.9)	
Procedure time (minutes), SD	448	585.1 ± 85.4	0.11
EBL, SD	200	466.5 ± 296.3	0.37
Bone segments, (%)			0.21
1	0 (0)	31 (36)	
2	0 (0)	39 (45.3)	
3	1 (100)	15 (17.4)	
4	0 (0)	1 (1.2)	
Tumor stage, (%)			0.94
pT_1_	0 (0)	8 (9.3)	
pT_2_	0 (0)	11 (12.8)	
pT_3_	0 (0)	5 (5.8)	
pT_4_	1 (100)	62 (72.1)	
Nodal stage, (%)			0.97
pN_0_	0 (0)	58 (67.4)	
pN_1_	1 (100)	11 (12.8)	
pN_2_	0 (0)	13 (15.1)	
pN_3_	0 (0)	4 (4.7)	
PNI present	1 (100)	23 (26.7)	0.28
LVI present	1 (100)	16 (18.6)	0.20
ENE present	0 (0)	7 (8.2)	1.00
Adjuvant therapy			0.70
None or declined	0 (0)	22 (25.6)	
Radiotherapy	1 (100)	50 (58.1)	
Chemoradiotherapy	0 (0)	14 (16.3)	
Follow-up (months)	21	38.5 ± 18.8	0.36
Clinical outcome			<0.01 *
NED	0 (0)	62 (72.1)	
AWD	0 (0)	15 (17.4)	
DOD	1 (100)	4 (4.7)	
DOO	0 (0)	5 (5.8)	

* Statistically significant (*p* < 0.05).

**Table 3:** Associations between CAS and bone margin status.

Study Variable	Positive Margin n = 1	Negative Margin n = 86	*p*-Value
Predictor variable, (%)			1.00
CAS	0 (0)	35 (40.7)	
Non-CAS	1 (100)	51 (59.3)	

**Table 4:** Associations between CAS and soft tissue margin status.

Study Variable	Positive Margin n = 3	Negative Margin n = 84	*p*-Value
Predictor variable, (%)			1.00
CAS	1 (33.3)	34 (40.5)	
Non-CAS	2 (66.7)	50 (59.5)	

**Table 5:** Subjects with positive bone or soft tissue margins.

Predictor	Age	Sex	pT	pN	Location	Re-Excision	Adjuvant Treatment	Recurrence
Non-CAS	73	F	2	0	Mandible ^§^	No	Declined	Locoregional
Non-CAS	62	M	4	1	Mandible ^§^	No	RT	Locoregional
Non-CAS	63	M	4	1	Mandible	No	CRT	Distant
CAS	62	F	4	1	Mandible ^§^	No	RT	Distant

^§^ Soft tissue margin.

**Table 6:** Association between predictor variables and local recurrence.

	Local Recurrence		
Study Variable	Yes (n = 21)	No (n = 66)	*p*-Value
Age (years), SD	63.2 ± 10.7	63.9 ± 10.9	0.79
Sex, (%)			0.82
Female	8 (38.1)	27 (40.9)	
Male	13 (61.9)	39 (59.1)	
ASA class, (%)			0.70
2	0 (0)	2 (3)	
3	20 (95.2)	60 (90.9)	
4	1 (4.8)	4 (6.1)	
BMI, SD	27 ± 5.1	25.9 ± 5.3	0.41
Tobacco status, (%)			0.17
Current/former	15 (71.4)	36 (54.5)	
Never	6 (28.6)	30 (45.5)	
Alcohol status, (%)			0.59
Current/former	11 (52.4)	39 (59.1)	
Never	10 (47.6)	27 (40.9)	
Time to treatment (days), SD	26.3 ± 13.7	30.3 ± 16	0.31
Jaw, (%)			0.72
Maxilla	2 (9.5)	10 (15.2)	
Mandible	19 (90.5)	56 (84.8)	
Flap, (%)			0.77
OC-RFFF	5 (23.8)	14 (21.2)	
FFF	16 (76.2)	52 (78.8)	
Procedure time (minutes), SD	582.3 ± 71.5	583.9 ± 90.8	0.94
EBL, SD	481.6 ± 342	457.6 ± 283.2	0.76
Bone segments, (%)			0.94
1	7 (33.3)	24 (36.4)	
2	10 (47.6)	29 (43.9)	
3	4 (19)	12 (18.2)	
4	0 (0)	1 (1.5)	
Tumor stage, (%)			0.77
pT_1_	1 (4.8)	7 (10.6)	
pT_2_	2 (9.5)	9 (13.6)	
pT_3_	1 (4.8)	4 (6.1)	
pT_4_	17 (81)	46 (69.7)	
Nodal stage, (%)			0.27
pN_0_	14 (66.7)	44 (66.7)	
pN_1_	4 (19)	8 (12.1)	
pN_2_	1 (4.8)	12 (18.2)	
pN_3_	2 (9.5)	2 (3)	
Stage			0.79
I	1 (4.8)	7 (10.6)	
II	2 (9.5)	4 (6.1)	
III	1 (4.8)	7 (10.6)	
IV	16 (76.2)	46 (69.7)	
IVb	1 (4.8)	2 (3)	
PNI present	7 (33.3)	14 (66.7)	0.50
LVI present	4 (19)	13 (19.7)	1.00
ENE present	2 (10)	5 (7.6)	0.66
Adjuvant therapy			0.20
None or declined	5 (23.8)	17 (25.8)	
Radiotherapy	10 (47.6)	41 (62.1)	
Chemoradiotherapy	6 (28.6)	8 (12.1)	
Chemotherapy	0 (0)	2 (2.7)	
Follow-up (months)	34.2 ± 23.3	39.6 ± 17.1	0.25
Clinical outcome			<0.01 *
NED	6 (28.6)	56 (84.8)	
AWD	11 (52.4)	4 (6.1)	
DOD	4 (11)	1 (1.5)	
DOO	0 (0)	5 (7.6)	

* Statistically significant (*p* < 0.05).

**Table 7:** Association between CAS and local recurrence.

	Local Recurrence		
	Yes (n = 21)	No (n = 66)	*p*-Value
Predictor variable			0.08
CAS, (%)	5 (23.8)	30 (45.5)	
Non-CAS, (%)	16 (76.2)	36 (54.5)	

## Data Availability

The raw data supporting the conclusions of this article will be made available by the authors on request.

## Abbreviations

CAS	Computer-aided surgery
SD	Standard deviation
ASA	American Society of Anesthesiologists
OC-RFFF	Osteocutaneous radial forearm free flap
FFF	Fibular free flap
PNI	Perineural invasion
LVI	Lymphovascular invasion
ENE	Extranodal extension
NED	No evidence of disease
AWD	Alive with disease
DOD	Died of disease
DOO	Died of other causes
RT	Radiation therapy
CRT	Chemoradiotherapy
